# Development and validation of prognostic nomograms in patients with gallbladder mucinous adenocarcinoma: A population-based study

**DOI:** 10.3389/fonc.2022.1084445

**Published:** 2022-12-14

**Authors:** Xiaoming Xu, Jingzhi Wang

**Affiliations:** ^1^ Department of Gastroenterology, Jining First People’s Hospital, Jining, China; ^2^ Department of Radiotherapy Oncology, The Affiliated Yancheng First Hospital of Nanjing University Medical School, The First People’s Hospital of Yancheng, Yancheng, China

**Keywords:** nomogram, gallbladder mucinous adenocarcinoma, SEER database, cancer-specific survival, overall survival

## Abstract

**Background:**

Gallbladder mucinous adenocarcinoma (GBMAC) is an uncommon malignant gallbladder tumor. There are few studies on its prognosis, with the majority consisting of small series or individual cases. We sought to develop and validate nomograms for predicting overall survival (OS) and cancer-specific survival (CSS) in GBMAC patients.

**Methods:**

The clinicopathological data of GBMAC patients from 1975 to 2019 was extracted from the Surveillance, Epidemiology, and End Results (SEER) database, and all patients were randomly divided into a training cohort (70%) and a validation cohort (30%). Using multivariate Cox regression analyses based on Akaike information criterion (AIC), prognostic and important variables for GBMAC were determined. On the basis of these factors, nomograms were developed to predict the 1-, 3-, and 5-year OS and CSS rates of patients with GBMAC. Multiple parameters, including the area under the subject operating characteristic curve (AUC), the calibration plots, and the decision curve analysis (DCA), were then used to evaluate the accuracy of nomograms.

**Results:**

Following exclusion, a total of 707 GBMAC patients were enrolled, and the training cohort (490, 70%) and validation cohort (217, 30%) were randomly assigned. Grade, surgery, radiation, and SEER stage were predictive factors for patients with GBMAC, as indicated by univariate and multivariate Cox regression analyses based on AIC. We created nomograms for predicting OS and CSS in GBMAC using the four factors. The calibration curves and area under the curves (AUCs) indicated that our nomograms have a moderate degree of predictive accuracy and capability. The results of the DCA revealed that the nomogram has a high predictive value.

**Conclusion:**

We established the first nomograms for predicting 1-, 3-, and 5-year OS and CSS in GBMAC patients, thereby contributing to the prognostication of patients and clinical management.

## 1 Introduction

Gallbladder cancer (GBC) is a highly fatal disease with a poor prognosis. It is the most common malignancy of the biliary system and the fifth most common malignancy of the digestive system (1). There are significant ethnic and geographic differences in the incidence of gallbladder cancer, which is higher in Chile and some Asian countries, and has a high mortality rate (2). Radical surgery is the only treatment modality that may completely cure gallbladder cancer, but because of the lack of specific clinical symptoms, many patients are already at an advanced stage at diagnosis, thus losing the opportunity for surgical treatment. The prognosis of patients with gallbladder cancer remains poor, although several new diagnostic and prognostic biomarkers have been identified in recent years (3, 4). The overall average survival rate for patients with GBC is 6 months and the 5-year survival rate is 5% (5).

Mucinous adenocarcinomas (MAs) are a rare pathological subtype of adenocarcinoma in which more than 50% of the total tumor volume is extracellular mucinous component (6). In general, the abundance of mucin disrupts intercellular interactions and promotes cell growth independent of apposition, thus creating the necessary conditions for metastasis and invasion (7). However, the prognostic value of MAs compared to non-MAs is controversial. Several studies have shown that mucinous adenocarcinoma of the colorectum has a poor prognosis (8, 9), but in the breast mucinous adenocarcinoma has a better prognosis than invasive ductal carcinoma (10), and some studies have shown no significant difference in prognosis between patients with mucinous and non-mucinous cancers (11, 12). Gallbladder mucinous adenocarcinoma (GBMAC) is one of the rarest subtypes of gallbladder cancer, accounting for less than 5% of case reports (13). There are very few studies on the clinical features and prognosis of GBMAC, which are limited to some case reports and small retrospective studies (14–16). Because of its rarity, its clinicopathological features and prognosis have not been well explored so far.

Nomograms are digital graphical tools that integrate a number of important characteristics and are increasingly frequently used for event prediction, particularly for cancer prognostic prediction. Several nomograms have been developed for the prognosis and treatment of patients with gallbladder cancer (17–19). However, due to the rarity of GBMAC, no nomograms that predict overall survival (OS) or cancer-specific survival (CSS) have been created so far.

SEER database is a comprehensive and authoritative online source that collects and integrates cancer data of about 34.6% of the United States population (20). Therefore, in this present study, based on data from the SEER database, we aimed to identify clinicopathologic characteristics of GBMAC, then constructed and validated OS and CSS nomograms for GBMAC patients.

## 2 Materials and methods

### 2.1 Data source and data collection

Data for the GBMAC between 1975 and 2019 were extracted from the SEER database using SEER*Stat software (version 8.4.0.1). All of our data for this study are available at http://seer.cancer.gov/. Since the SEER database is a publicly accessible database and patient information is anonymized, no ethical review is necessary for our study.

**Figure 1:**
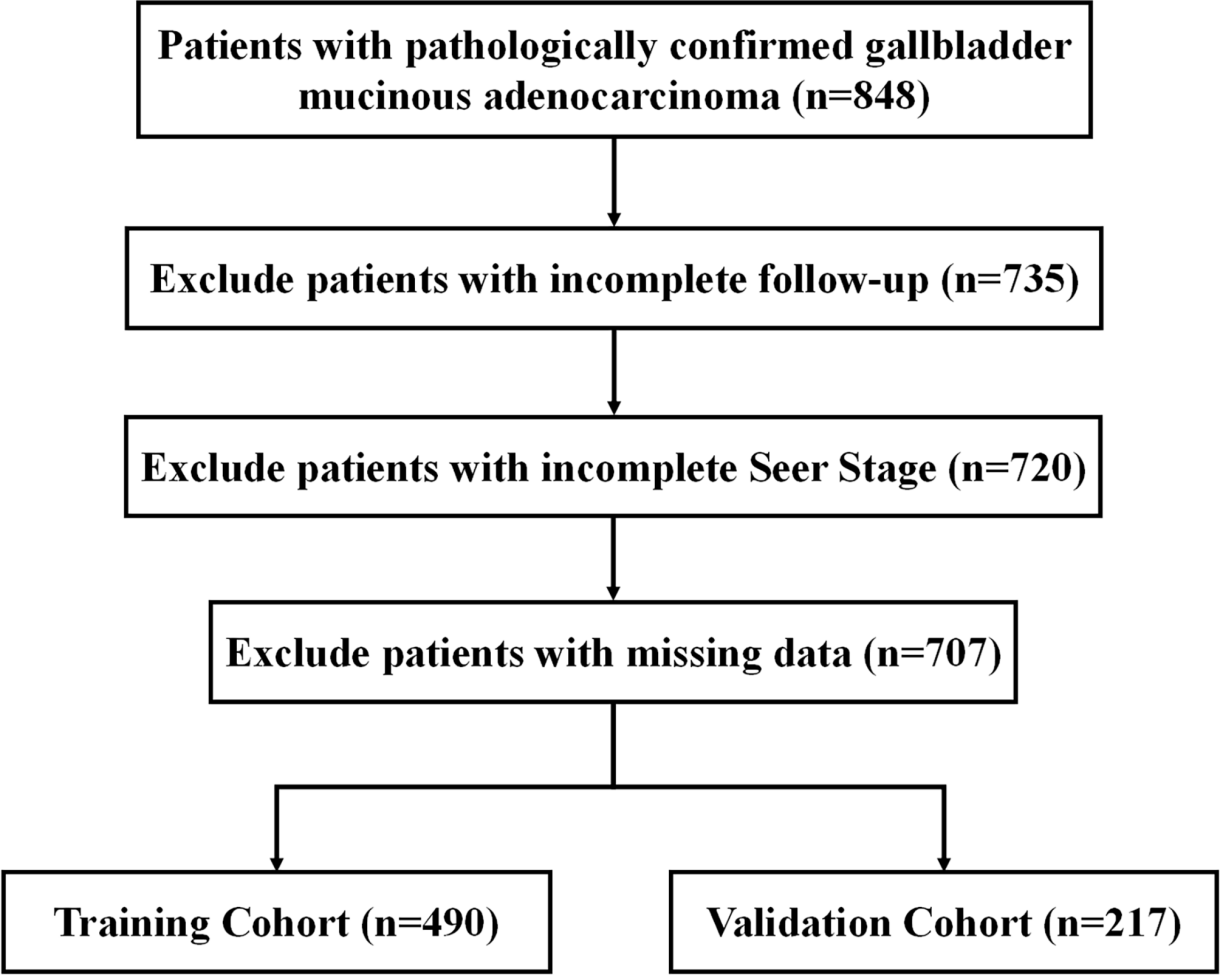
Workflow for inclusion and exclusion of patients with GBMAC.

Inclusion criteria were patients with pathologically confirmed primary malignant mucinous adenocarcinoma, primary site code (C23.9) and Third Edition 3 (ICD-O-3) histology codes (8480/3 and 8481/3). We then excluded patients with incomplete follow-up information, unknown SEER stage and missing data. The flowchart is demonstrated in **Figure 1**.

Age, gender, marital status, grade, SEER stage, treatment (surgery, chemotherapy, radiotherapy), vital state, survival time, and cause of death were collected from the database. OS was defined as the duration from the time of diagnosis and death or the final follow-up. CSS was defined as the period between diagnosis and death from gallbladder mucinous adenocarcinoma or the final follow-up.

### 2.2 Nomograms construction and validation

Utilizing the univariate Cox regression analysis, variables related with survival were determined. Based on the smallest Akaike information criterion (AIC) in the multivariate Cox regression analyses, prognostic and important variables for GBMAC were determined. On the basis of these factors, nomograms were developed to predict the 1-, 3-, and 5-year OS and CSS rates of patients with GBMAC. The capacity of nomograms to make accurate predictions was demonstrated with the help of calibration curves. In this study, time-dependent receiver operating characteristic (ROC) curves were utilized in order to assess the discriminatory power of nomograms. The previously established nomograms were applied to the validation cohort in order to validate them, and the associated analyses were carried out once more.

### 2.3 Clinical associations

The clinical utility of the nomograms was evaluated using decision curve analysis (DCA). Using the nomograms, the optimal risk score cutoff value for each patient was determined, and all patients in the training and validation cohorts were divided into high-risk and low-risk groups. Following this, we examined CSS and OS across the two risk groups using K-M survival curves in both the training cohort and validation cohort to examine any variations in survival.

### 2.4 Statistical analysis

Chi-square or nonparametric U tests were used to compare groups. Chi-square was utilized to compare the frequency (percentage) of different variable groups. Using the log-rank test and K-M curves, differences in group survival were investigated. All statistical analyses were performed using R (version 3.6.2) software. A P-value of 0.05 or less was regarded as statistically significant throughout this study.

## 3 Results

### 3.1 Clinicopathological characteristics of patients

Between 1975 and 2019, 707 individuals with GBMAC were registered in the primary cohort before being randomly assigned to the training cohort (490, 70%) and validation cohort (217, 30%). **Table 1** summarizes the clinicopathological features of individuals participating in both the training cohort and the validation cohort. 65.2% of patients are older than 65, while 34.8% are younger than 65. Females represented 69.4% of all patients, while men represented 30.6%. Patients who did not get married (47.8%) were comparable to those who did get married (48.7%). Regarding grade, the majority are in grades I-II (45.0%). Regarding SEER stage, the majority were distant (44.8%), while the rest were regional (29.6%) and localized (2.4%). (25.6). 72.3% underwent surgery, whereas 27.7% do not. A minority of patients (11.3%) had radiation, while the majority (84.0%) did not. In addition, patients who received chemotherapy (33.8%) were much less than those who did not (66.2%). These clinicopathological variables did not differ significantly between the training cohort and the validation cohort (all P > 0.05).

**Table 1:** Clinicopathological characteristics of patients with GBMAC.

Characteristics	Level	Overall	Validation Cohort	Training Cohort	*P-*value
		n=707	n=217	n=490	
**Age**	<65	246(34.8)	80(36.9)	166(33.9)	0.494
	≥65	461(65.2)	137(63.1)	324(66.1)	
**Gender**	Female	491(69.4)	147(67.7)	344(70.2)	0.571
	Male	216(30.6)	70(32.3)	146(29.8)	
**Marital**	No	338(47.8)	101(46.5)	237(48.4)	0.840
	Unknown	25(3.5)	7(3.2)	18(3.7)	
	Yes	344(48.7)	109(50.2)	235(48.0)	
**Grade**	I-II	318(45.0)	104(47.9)	214(43.7)	0.415
	III-IV	163(23.1)	51(23.5)	112(22.9)	
	Unknown	226(32.0)	62(28.6)	164(33.5)	
**Seer Stage**	Distant	317(44.8)	93(42.9)	224(45.7)	0.473
	Localized	181(25.6)	53(24.4)	128(26.1)	
	Regional	209(29.6)	71(32.7)	138(28.2)	
**Chemotherapy**	No/Unknown	468(66.2)	133(61.3)	335(68.4)	0.080
	Yes	239(33.8)	84(38.7)	155(31.6)	
**Radiotherapy**	No/Unknown	594(84.0)	185(85.3)	409(83.5)	0.627
	Yes	113(16.0)	32(14.7)	81(16.5)	
**Surgery**	No/Unknown	196(27.7)	55(25.3)	141(28.8)	0.396
	Yes	511(72.3)	162(74.7)	349(71.2)	

GBMAC, Gallbladder mucinous adenocarcinoma.

### 3.2 Construction of nomograms to predict OS and CSS at 1-, 3-, 5-year

**Figure 2:**
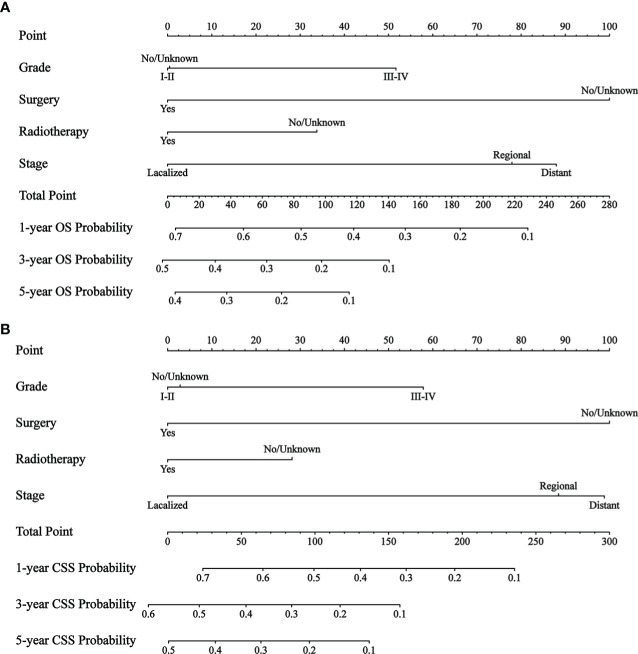
The nomograms to predict OS and CSS at 1-, 3-, 5-year for patients with GBMAC. **(A)** The nomogram to predict OS for patients with GBMAC. **(B)** The nomogram to predict CSS for patients with GBMAC.

By univariate COX regression analysis, we found that grade, marital status, SEER stage, radiotherapy, and surgery were significant factors for OS (**Table 2**). These factors were significant associated with CSS except marital status (**Table 3**). The smallest Akaike information criterion (AIC) value occurred when we incorporated 4 factors (grade, SEER stage, radiotherapy, and surgery) into the multivariate Cox regression model for OS (AIC = 4521.3) and CSS (AIC=4121.1). Then those prognostic factors were applied to construct two different nomograms for OS and CSS prediction at 1-, 3-, and 5-year, respectively (**Figure 2**). As shown in the nomograms, grade, SEER stage, radiotherapy, and surgery were essential prognostic predictors for OS and CSS. Surgery was the most influential risk factor for both OS and CSS according to the nomograms. SEER stage was also important factors influence the patient survival.

**Table 2:** Univariate and multivariate Cox regression analysis of OS in training cohort.

Characteristics	Univariate analysis		Multivariate analysis	
	HR (95%CI)	*P* value	HR (95%CI)	*P* value
Age				
<65	Reference			
≥65	1.182(0.966-1.445)	0.104		
Gender				
Female	Reference			
Male	1.061(0.864-1.303)	0.574		
Marital status				
No	Reference			
Yes	0.974(0.804-1.181)	0.789		
Unknown	0.569(0.325-0.997)	0.049		
Grade				
I-II	Reference			
III-IV	1.648(1.294-2.099)	<0.001	1.540(1.205-1.969)	0.001
Unknown	1.527(1.224-1.905)	<0.001	1.004(0.791-1.274)	0.974
Seer Stage				
Distant	Reference			
Localized	0.36(0.282-0.459)	<0.001	0.479(0.366-0.628)	<0.001
Regional	0.733(0.586-0.918)	0.007	0.92(0.726-1.164)	0.486
Chemotherapy				
No/Unknown	Reference			
Yes	0.872(0.71-1.072)	0.194		
Radiotherapy				
No/Unknown	Reference			
Yes	0.734(0.568-0.949)	0.018	0.754(0.579-0.981)	0.036
Surgery				
No/Unknown	Reference			
Yes	0.35(0.282-0.434)	<0.001	0.434(0.338-0.556)	<0.001

OS, Overall survival; HR, Hazard ratio.

**Table 3:** Univariate and multivariate Cox regression analysis of CSS in training cohort.

Characteristics	Univariate analysis		Multivariate analysis	
	HR (95%CI)	*P* value	HR (95%CI)	*P* value
Age				
<65	Reference			
≥65	1.094(0.888-1.349)	0.399		
Gender				
Female	Reference			
Male	1.048(0.843-1.303)	0.672		
Marital status				
No	Reference			
Yes	1.003(0.819-1.227)	0.977		
Unknown	0.608(0.339-1.09)	0.095		
Grade				
I-II	Reference			
III-IV	1.779(1.382-2.29)	<0.001	1.665(1.289-2.15)	<0.001
Unknown	1.61(1.276-2.033)	<0.001	1.025(0.798-1.318)	0.845
Seer Stage				
Distant	Reference			
Localized	0.305(0.233-0.398)	<0.001	0.418(0.312-0.561)	<0.001
Regional	0.717(0.569-0.903)	0.005	0.913(0.715-1.164)	0.461
Chemotherapy				
No/Unknown	Reference			
Yes	0.921(0.744-1.14)	0.45		
Radiotherapy				
No/Unknown	Reference			
Yes	0.762(0.583-0.994)	0.045	0.78(0.593-1.027)	0.077
Surgery				
No/Unknown	Reference			
Yes	0.327(0.263-0.408)	<0.001	0.414(0.32-0.536)	<0.001

CSS, Cancer-specific survival; HR, Hazard ratio.

### 3.3 Validation of nomograms

**Figure 3:**
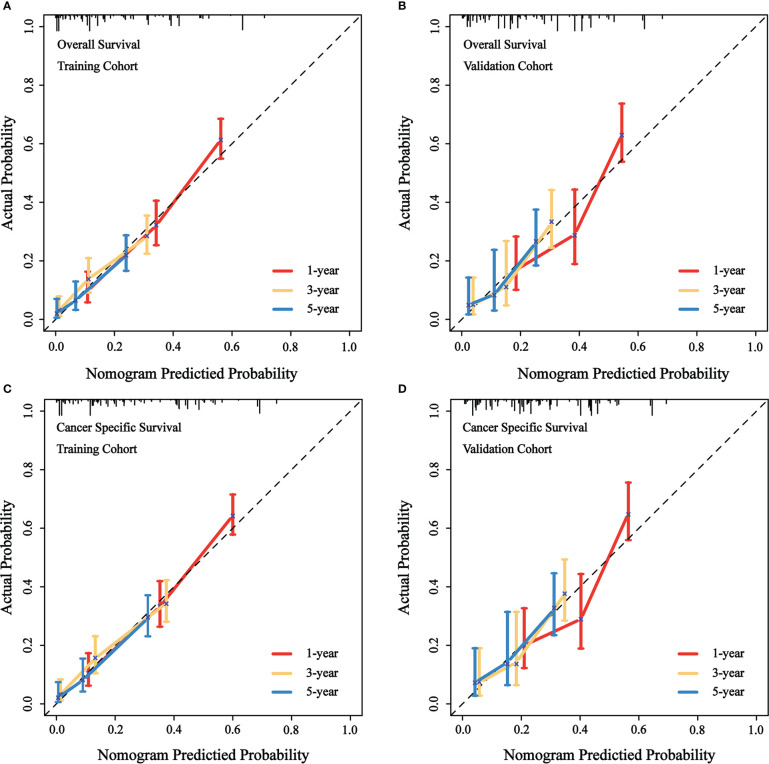
Calibration curves of the nomograms to predict OS and CSS at 1-, 3-, 5-year for patients with GBMAC. **(A)** Calibration curve of the nomogram to predict OS at 1-, 3-, 5-year in training cohort. **(B)** Calibration curve of the nomogram to predict OS at 1-, 3-, 5-year in validation cohort. **(C)** Calibration curve of the nomogram to predict CSS at 1-, 3-, 5-year in training cohort. **(D)** Calibration curve of the nomogram to predict CSS at 1-, 3-, 5-year in validation cohort. The horizontal axis of the nomogram represents the expected value, while the vertical axis represents the observed value.

**Figure 4:**
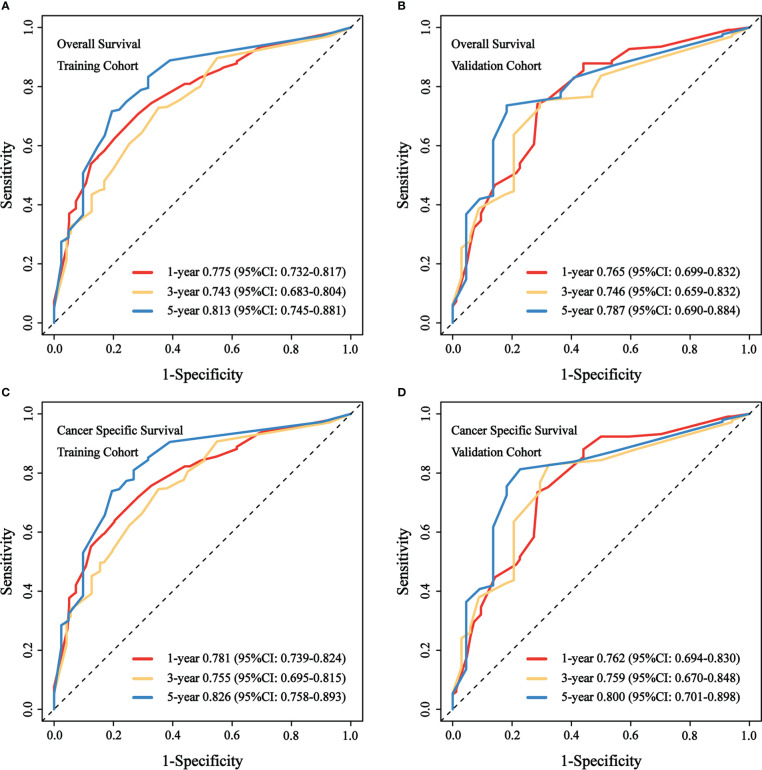
Time-dependent ROC curves to predict OS and CSS at 1-, 3-, 5-year for patients with GBMAC. **(A)** AUCs at 1-, 3-, 5-year for OS prediction in training cohort were 0.775, 0.734, 0.813. **(B)** AUCs at 1-, 3-, 5-year for OS prediction in validation cohort were 0.765, 0.746, 0.787. **(C)** AUCs at 1-, 3-, 5-year for CSS prediction in training cohort were 0.781, 0.755, 0.826. **(D)** AUCs at 1-, 3-, 5-year for CSS prediction in validation cohort were 0.762, 0.759, 0.800.

We evaluated the predictive performance of the nomograms through calibration curves and time-dependent ROC curves. As shown in **Figure 3**, the predicted survival probabilities for OS and CSS (calibration curves) were highly linear to the actually observed survival probabilities in both the training cohort and validation cohort. This indicated robust predictive accuracy of our nomograms. Subsequently, time-dependent ROC curves for OS and CSS in both the training cohort and validation cohort were depicted with corresponding AUCs calculated (**Figure 4**). In training cohort, AUCs at 1-, 3-, 5-year were 0.775, 0.734, 0.813 for OS and 0.781, 0.755, 0.826 for CSS. In validation cohort, AUCs at 1-, 3-, 5-year were 0.765, 0.746, 0.787 for OS and 0.762, 0.759, 0.800 for CSS. This suggested that our nomograms are well capable of discrimination.

### 3.4 Clinical application of nomograms

**Figure 5:**
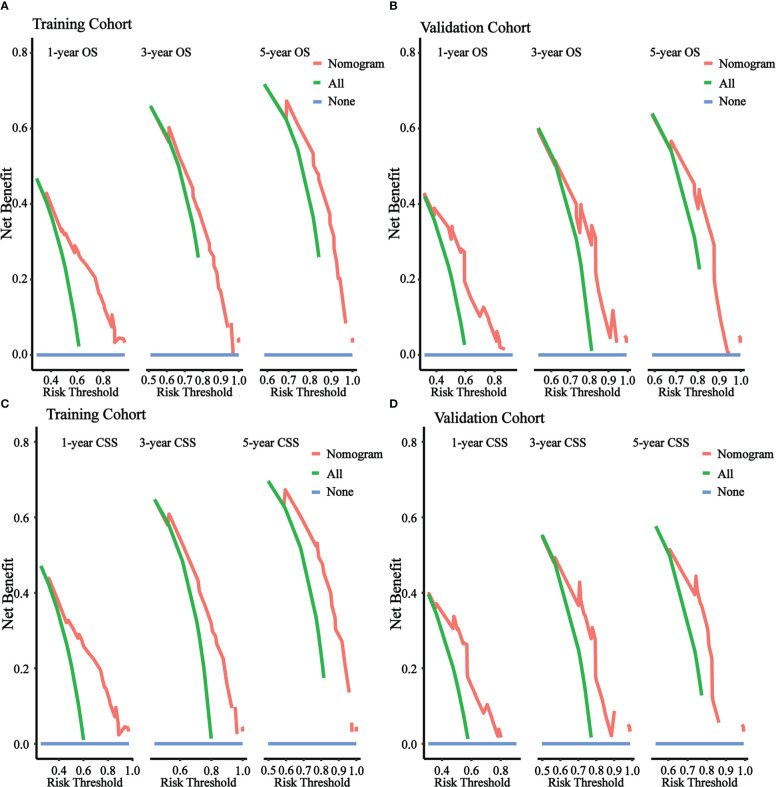
DCA of the nomograms to predict OS and CSS. **(A)** DCA of the nomogram to predict OS at 1-, 3-, 5-year in training cohort. **(B)** DCA of the nomogram to predict OS at 1-, 3-, 5-year in validation cohort. **(C)** DCA of the nomogram to predict CSS at 1-, 3-, 5-year in training cohort. **(D)** DCA of the nomogram to predict CSS at 1-, 3-, 5-year in validation cohort.

**Figure 6:**
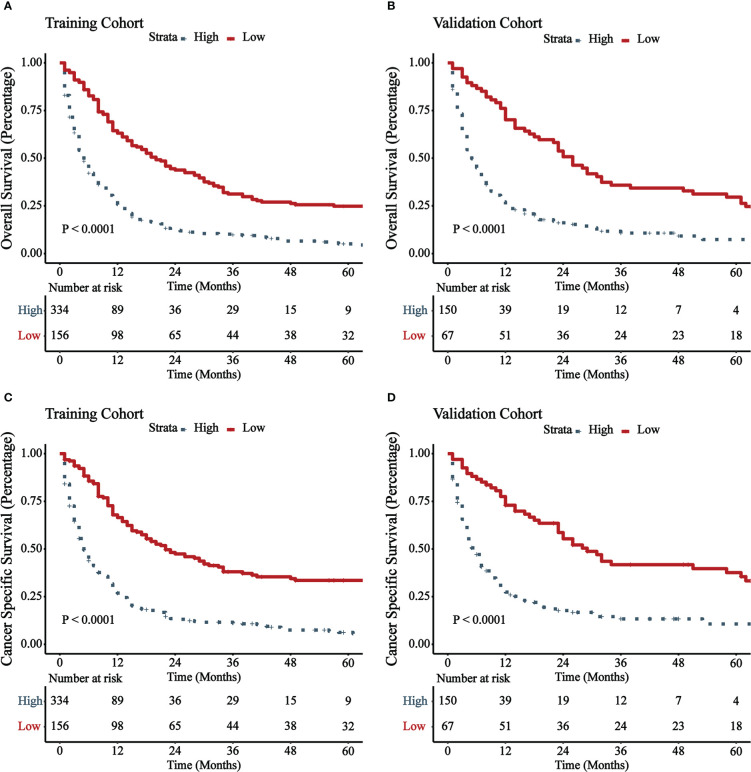
K-M survival curves of patients with GBMAC in high-risk group and low-risk group. **(A)** OS comparison of patients with GBMAC based on risk score grouping in training cohort. **(B)** OS comparison of patients with GBMAC based on risk score grouping in validation cohort. **(C)** CSS comparison of patients with GBMAC based on risk score grouping in training cohort. **(D)** CSS comparison of patients with GBMAC based on risk score grouping in validation cohort.

**Figure 5** illustrated the results of DCA. In both the training cohort and the validation cohort, it demonstrated clinical utility. Used the ROC curve, the risk score and optimal cut-off value for each patient were calculated based on the nomograms. For comparison of OS, patients were divided into high-risk (total score 88.43) and low-risk (total score 88.43) groups. For CSS comparison, patients were divided into high-risk (total score 101.67) and low-risk (total score 101.67) groups. The K-M survival curves demonstrated that patients in the high-risk group have a significantly worse prognosis (both OS and CSS) in both the training cohort and validation cohort (all P < 0.0001, **Figure 6**). For OS, the 1-, 3-, and 5-year predicted survival probabilities for the high-risk group were 25.8%, 10.1%, and 5.7%, while those for the low-risk group were 65.2%, 32.6%, and 26.2%. The 1-, 3-, and 5-year predicted survival probabilities for CSS were 27.0%, 11.9%, and 7.5% for the high-risk group and 68.2%, 32.6%, and 34.4% for the low-risk group.

## 4 Discussion

Based on a large population from the SEER database, we were able to successfully design two nomograms to predict OS and CSS at 1, 3, and 5 years for patients with GBMAC. The prediction accuracy and capabilities of our nomograms were validated further in both the training cohort and validation cohort. A number of important prognostic indicators were identified and included in our nomograms. OS and CSS prediction factors include grade, SEER stage, radiation, and surgery. The GBMAC prediction model that we built contributes to the understanding of the features of GBMAC patients and clinical decision-making.

Nomograms are utilized as a visual numerical graphical tool to forecast the probability of occurrence of a particular event based on data with known factors. In recent years, nomograms have been widely used to predict the prognosis of various cancers, such as esophageal, pancreatic, breast and prostate cancers (21–24). For gallbladder cancer, several nomograms with different predictive functions have also been developed (18, 25, 26). However, because the GBMAC is so rare, most of the current research on it comes from case reports and small cohort studies. Limited evidence suggests that it is more advanced at the time of diagnosis and exhibits a more aggressive and poorer survival outcome compared to common adenocarcinoma (14, 16). As a result, it is necessary to develop a reliable prognostic model in order to make accurate predictions regarding the outcome of GBMAC.

Our research revealed that the incidence was higher in older and female patients, that the vast majority of patients had local or distant metastases at the time of diagnosis, that the majority of patients underwent surgery, and that the proportion of patients receiving chemotherapy and radiotherapy was relatively low. Grade, SEER stage, surgery, and radiation were found to be important risk factors affecting patients’ OS and CSS. The only effective curative treatment for those with gallbladder cancer is surgical resection. The majority of patients were treated with surgery, which was also a predictor of a good prognosis, according to our study. Regarding its use as an adjuvant therapy for people with gallbladder cancer, radiotherapy is still debatable. Radiotherapy is not the primary treatment for patients with gallbladder cancer, it can be an effective adjuvant treatment for certain specific patient groups, especially those at high risk of recurrence such as R1 resection or lymph node positivity. However, the general condition of the patient needs to be evaluated before radiotherapy. A previous study showed that in patients with gallbladder cancer who underwent radical resection, radiation provided a short-term survival benefit (27). An earlier study showed that adjuvant radiotherapy improved survival in patients with gallbladder cancer with regional lymph node metastasis (28). Our study also showed that radiotherapy was a protective factor for patient prognosis. Poorly differentiated pathological types imply a more aggressive nature, while local metastases or distant metastases imply a loss of access to surgical treatment and a higher risk of recurrence, often implying a poorer prognosis. Grade and SEER stage have been identified as risk factors in several previously established models for predicting the prognosis of patients with gallbladder cancer (29, 30). The same results were obtained in our current study that grade and SEER stage were the important risk factors. Based on the scores obtained from the nomograms of OS and CSS, we classified the patients into high-risk and low-risk groups, respectively. The K-M curves revealed additional evidence of a statistically significant difference in the amount of time spent alive between these two groups. Consequently, if the nomogram recognizes a patient whose OS score or CSS score is in the high-risk group, the clinician can be on the lookout for this patient and make timely adjustments to the follow-up treatment methods, such as implementing combined modality therapy as soon as possible, to help the patient achieve a better curative effect. This will serve as a reference for clinicians, allowing them to make decisions on their work more effectively.

However, this research has some limitations. First, all of our data came from the SEER database, which includes patients from the United States. Validation is required to determine whether it can be used for prognostic analysis of all patients, and it may require external multicenter validation. Second, the accuracy of the prediction model may be impacted by incomplete data on several variables in the SEER database, such as chemotherapy regimen and radiation dose. Finally, due to the large time gap between the earliest and most recent patients included in this study, changes in detection tests and treatment modalities may have had an impact on patient survival rates.

## 5 Conclusions

We constructed new nomograms to predict OS and CSS at 1-, 3-, and 5-year for patients with GBMAC. The nomograms were evaluated with robust predictive accuracy and capability whereby to contribute to clinical management and risk decisions.

## Data availability statement

The original contributions presented in the study are included in the article/supplementary material. Further inquiries can be directed to the corresponding author.

## Ethics statement

The data of this study is obtained from the SEER database. The patients’ data is public and anonymous, so this study does not require ethical approval and informed consent.

## Author contributions

XX and JW designed the study. XX collected and analyzed the data. XX drafted the initial manuscript. JW reviewed and edited the article. All authors approved the final manuscript. All authors contributed to the article and approved the submitted version.
